# Glutathione in Cancer Cell Death

**DOI:** 10.3390/cancers3011285

**Published:** 2011-03-11

**Authors:** Angel L. Ortega, Salvador Mena, Jose M. Estrela

**Affiliations:** 1 Department of Physiology, Faculty of Medicine and Odontology, University of Valencia, 17 Av. Blasco Ibanez, 46010 Valencia, Spain; E-Mail: angel.ortega@uv.es; 2 Green Molecular SL, Pol. Ind. La Coma-Parc Cientific, 46190 Paterna, Valencia, Spain; E-Mail: salvador.mena@uv.es

**Keywords:** glutathione, cancer, cell death, apoptosis, necrosis, autophagy

## Abstract

Glutathione (L-*γ*-glutamyl-L-cysteinyl-glycine; GSH) in cancer cells is particularly relevant in the regulation of carcinogenic mechanisms; sensitivity against cytotoxic drugs, ionizing radiations, and some cytokines; DNA synthesis; and cell proliferation and death. The intracellular thiol redox state (controlled by GSH) is one of the endogenous effectors involved in regulating the mitochondrial permeability transition pore complex and, in consequence, thiol oxidation can be a causal factor in the mitochondrion-based mechanism that leads to cell death. Nevertheless GSH depletion is a common feature not only of apoptosis but also of other types of cell death. Indeed rates of GSH synthesis and fluxes regulate its levels in cellular compartments, and potentially influence switches among different mechanisms of death. How changes in gene expression, post-translational modifications of proteins, and signaling cascades are implicated will be discussed. Furthermore, this review will finally analyze whether GSH depletion may facilitate cancer cell death under *in vivo* conditions, and how this can be applied to cancer therapy.

## 1. Glutathione in Cancer Biology

Normal cells respond to external stimuli by different tightly regulated pathways. However, in cancer cells, physiology is altered leading to, e.g., excessive growth and reduced cell death. Classically, the loss of growth regulation has been attributed to the mutation of oncogenes and tumor suppressor genes. Besides, reduction in the response to cell death stimuli is related to aberrant anti-death and pro-death protein expression [1].

Glutathione (GSH, γ-glutamyl-cysteinyl-glycine), is the most abundant non-protein thiol in eukaryotic cells. The synthesis of this ubiquitous tripeptide is catalyzed by two cytosolic enzymes: γ-glutamate-cysteine ligase (first step), and GSH synthetase (second step; which combines γ-glutamyl-cysteine with glycine to generate GSH). Cys availability and γ-glutamate-cysteine ligase activity are the rate-limiting factors in GSH synthesis [2]. Owing to its reactivity and high intracellular concentrations (approx. 10 mM in the liver and different malignant cells), GSH is involved in cell protection against free radicals, and in many cellular functions being particularly relevant in regulating carcinogenic mechanisms [3,4]; sensitivity against xenobiotics, ionizing radiation and some cytokines [5-11]; DNA synthesis; and cell proliferation [2,12-14] .

GSH biochemistry deregulation in tumors has been observed in many different murine and human cancers [15-17]. In addition to the properties mentioned above, particularly in cancer cells, GSH is important in the protection against tumor microenvironment-related aggression [18], apoptosis evasion [19], colonizing ability [20], and multidrug and radiation resistance [2,7-9,11,20-23]. Increased levels of GSH and resistance to chemotherapeutic agents have been observed, e.g., for platinum containing compounds, alkylating agents (such as melphalan), anthracyclines, doxorubicin, and arsenic [24].

Modifications of GSH metabolism and the introduction of agents able to modulate GSH concentration in tumor cells opened up the possibility of regulating the cellular response to different anticancer treatments [9,10,23,25,26]. Nevertheless, approaches in cancer treatment based on modulating GSH levels appear highly limited by potential harmful effects to normal cells.

## 2. Redox Control of Cell Death

In mammals, under physiological conditions, the equilibrium between cell death and division helps to maintain tissue/organ homeostasis. Mechanisms related to this equilibrium involve cell cycle checkpoints, DNA repair and recombination, and cell death. In all these mechanisms the oxidation and reduction of proteins, as well as the rate and nature of free radicals generation, play important roles.

The term cellular redox state is classically used to describe the balance of NAD^+^/NADH, NADP^+^/NADPH, and/or GSH/GSSG, and its relationship to different sets of metabolites and the control of cell metabolism [27,28].

Free radicals are defined as molecules or fragments of molecules containing one or more unpaired electrons [29,30]. Highly reactive species capable of damaging carbohydrates, lipids, proteins and/or nucleic acids, and of causing loss of molecular functions [31].

Reactive oxygen species (ROS) represent the more abundant free radicals in mammalian cells [32] and include, mainly, superoxide anions (O_2_^·−^) [33], hydroxyl radicals (^·^OH)[34] and peroxide radicals (ROO^·^) [35]. One should also take into account the levels of other molecules, without unpaired electrons but also harmful and ROS-related, such as hydrogen peroxide (H_2_O_2_) [36]. Besides reactive nitrogen species, such as nitric oxide (·NO) [37] and the peroxynitrite (ONOO^−^)[38] also play essential regulatory roles. The sources of ROS are the electron transport system in the mitochondria, the Krebs cycle, different oxidases (including NADPH oxidase, xanthine oxidase, and certain arachidonic acid oxygenase activities) and the radicals released from immune cells [39-42].

**Figure 1.**
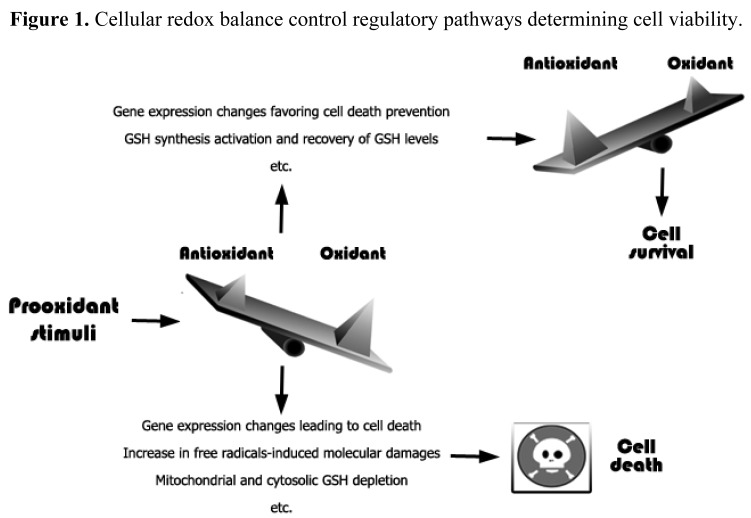
Cellular redox balance control regulatory pathways determining cell viability. Different effectors can lead to redox system oxidations triggering a plethora of cellular responses. If stimulation does not compromise cell resistance mechanisms negative feed-back systems restore the redox homeostasis and allow cell survival. However, if stimuli cannot be counteracted by the antioxidant machinery, redox status alterations cause irreversible loss of cell viability.

An increase in free radicals levels may lead to an increase in different cellular defense systems or, if the damage is irreversible, to cell death [43] (Figure 1). Moreover, oxidative stress or redox status shifts may cause cell transition from quiescent to proliferative status, growth arrested or cell death activation according to the duration and extent of the redox imbalance [44].

Although several mechanisms of cell death have been characterized, their classification is difficult because more than one single mechanism can be activated by the same signal [45]. Main cell death mechanisms include: (a) apoptosis, a process of programmed cell death, characterized by cell shrinkage, chromatin condensation, caspases activation and DNA fragmentation [46]; (b) necrosis, an uncontrolled event caused by loss of cell homeostasis where cell volume increases [46], and (c) autophagy, where degradation of cellular components through the lysosomal machinery is observed [47].

Apoptosis can be triggered either through an intrinsic pathway, which involves procaspase-9 activation downstream of mitochondrial proapoptotic events; or through an extrinsic pathway, triggered by membrane receptors [such as Fas ligand or tumor necrosis factor-α (TNF-α)] without direct involvement of mitochondria-derived signals [48]. In both cases, the apoptotic machinery activates cysteine-aspartate proteases [49].

Cells possess antioxidant systems to control the redox state and, thereby, survival [43]. Antioxidant defenses include superoxide dismutases; catalase; Cys; thioredoxins; peroxiredoxins; sulfiredoxins; GSH, and enzymes involved in GSH homeostasis, such as GSH peroxidase, GSH reductase, glutaredoxins, and GSH transferases; vitamins; metal-complex proteins, *etc.* [40,43,50]. In consequence, down regulation of these antioxidant defenses can lead to increased ROS levels, redox status alterations [19], and cell damage; thus increasing the risk of developing pathologies such as cancer, neurodegeneration, *etc* [19].

Oxidative stress associates with carbohydrate, lipid, protein and DNA damages, which lead to cellular dysfunction and, eventually, cell death [42]. Moreover oxidative stress and/or changes in the intracellular redox status may affect nuclear chromatin remodeling (histone acetylation/deacetylation) and cause changes in gene expression [51]. Indeed, oxidative stress causes: single- and double-strand DNA fragmentation [52]; damages in mitochondria that decrease the transmembrane potential, and may associate to permeability alterations and facilitate the release of death-related molecular signals [53,54].

Oxidative stress is a known inducer of the transcription of specific genes involved in cell death [55], whereas GSH has been also postulated as potential regulate of gene transcription [56]. Moreover, different GSH-related enzyme activities are regulated by a redox-sensitive transcription factor, NF-E2 p45-related factor-2 (Nrf2) [57]. The oxidation of specific proteins containing thiols induces the release of Nrf-2, which then translocates to the nucleus, activating transcriptions through binding to antioxidant response elements (ARE) in the control regions of multiple detoxification-related genes [58]. Potential redox-sensitive transcription factors, such as Nfr-2, and how they affect gene expression represent an open field for research. In fact, several transcription factors are modulated through oxidation/reduction of critical Cys localized in their DNA binding domain, essentials for recognition of the binding site through electrostatic interactions with specific DNA bases [59]. Oxidation of these Cys residues results in changes in the inter- or intramolecular disulfide bonds affecting the tridimensional structure of the transcription factor and, in consequence, its function [59,60]. This modulation can upregulate or downregulate gene expression, for instance of NF-κB or p53 [50], or different receptor tyrosine kinases [phosphokinase C and mitogen-activated protein kinase (MAPK)] [19]. MAPKs, such as ERK, p38 and JNK, are central players in the mechanisms of stress induced apoptosis [19].

As well as in transcription factors, changes in the thiol-disulfide status affecting critical Cys in enzymes, receptors, or transport proteins, can be reversible or irreversible [19]. Reversible modifications of Cys, Met, Trp, and/or Tyr residues (via nitrosylation, hydroxylation, glutathionylation, or disulfide bond formation) may differentially affect protein function. Besides, glycerophospholipids and other lipids in plasma and organelle membranes are also major targets of oxidizing agents. Lipid oxidation-derived products such as malondialdehyde, 4-hydroperoxy-2-nonenal, 4-o-xo-2-nonenal, or 4-hydroxy-2-nonenal, can impair membrane functions, inactivate, membrane-bound receptors and enzymes, and increase permeability [30,51].

In response to stress, the thioredoxin/glutaredoxin complex induces the autophosphorylation and activation of apoptosis signal regulating kinase 1 (ASK1), which causes phosphorylation and activation of JNK and p38, both involved in apoptosis initiation [61].

In case of DNA damage, p53 mediates the response through initiation of DNA repair, cell cycle arrest, or activation of an apoptotic signaling cascade. p53 activity is regulated by posttranslational modifications, including phosphorylation, acetylation, ubiquitination, sumoylation, glutathionylation, cytoplasmic sequestration, *etc.* [42]. p53 modulates activation of proapoptotic genes, or induces apoptosis through transcription-independent mechanisms (e.g., by altering binding actitivities of Bcl-2 or Bax) [19,42].

Finally, it is worthy to remark that some differences among mitochondria, cytoplasm, and nuclei can be found. For instance, a possible translocation of GSH into the nucleus in response to acute oxidative stress has been suggested [62]. In addition, in mitochondria, complex I glutathionylation results in increased superoxide production, then leading to activation of redox signaling pathways and/or induction of cell death, depending upon the magnitude of modifications [42].

## 3. Glutathione and the Mechanisms of Cancer Cell Death

Alterations in cell death mechanisms are common in the pathophysiology of different human diseases including cancer, neurodegenerative or autoimmune disorders. The signaling pathways leading to cell death, and to programmed cell death type I or apoptosis in particular, have been extensively characterized. However, recent studies point out the importance of changes in the intracellular milieu affecting apoptosis and other types of cell death. GSH depletion, in particular, is a common feature preceding cell demise.

### 3.1. GSH Role in Apoptosis

Although the relationship between GSH and apoptosis is not fully understood, GSH is essential for cell survival and its depletion increases the cellular susceptibility to apoptosis [63]. High intracellular GSH levels have been related to apoptosis resistance [64,65], and GSH depletion has been shown either to induce or potentiate apoptosis [64,66,67]. Buthionine sulfoximine (BSO), a selective inhibitor of γ-GCS, induces GSH depletion without triggering apoptosis, but facilitates and potentiates the response to other cell death stimulus. For example, BSO potentiates death receptor-induced apoptosis in T cells [64,66], and increases the susceptibility to TNF-α treatment in Ehrlich-ascites-tumor-bearing mice [14]. Besides, GSH supplementation with GSH ester, or replenishment of GSH pools with *N*-acetyl-L-cysteine or *S*-adenosyl-methionine (both precursors of Cys), have been shown to be effective protectors against apoptosis [65,68,69].

The intrinsic apoptotic pathway is mainly activated by ROS, which induces opening of the mitochondrial permeability transition pore [70,71], which may cause the release of proapoptotic molecules, such as cytochrome c (an intermembrane space protein), to the cytosol. Mitochondrial release of cytochrome C is required for the formation of apoptosome and caspase activation, and different death-related signals may cause its release to the cytosol. However, it is important to point out that the mitochondrial permeability transition, which may occur either in apoptosis or in necrosis, is a sudden increase in the permeability of the inner mitochondrial membrane to solutes with molecular masses of up to 1,500 Da. This process is due to opening of a voltage- and Ca^2+^-dependent, cyclosporin A-sensitive, high conductance channel called the permeability transition pore [72]. The long-standing idea that the permeability transition pore may form at inner-outer membrane contact sites and that it may be constituted by the adenine nucleotide translocator in the inner mitochondrial membrane and the voltage-dependent anion channel in the outer mitochondrial membrane has not been confirmed by genetic ablation of these proteins [73-75]. As of today, however, it is not clear whether the outer mitochondrial membrane is necessary for the permeability transition to occur and what regulatory properties, if any, it may contribute to the permeability transition pore. Among the variety of effectors that regulate the permeability transition pore open-closed transitions, oxidizing agents have received considerable attention, and changes in the redox state of pyridine nucleotides, GSH, and sulfhydryl groups have been shown to play a prominent regulatory role [76-81].

Apoptosome complexation, results in activation of caspase-9, which in turn activates effector caspases to carry out the process of apoptosis [40,82]. Opening of the mitochondrial permeability transition pore is facilitated by direct depletion of the mitochondrial GSH (mtGSH), even in the absence of high (non-physiological) ROS levels [83]. This fact indicates a direct regulatory role of mtGSH in regulating the first step in the mitochondria-dependent apoptotic cascade.

Furthermore, cellular GSH depletion induced by either abolishment of the γ-glutamate-cysteine ligase activity in knock-out mice, Cys starvation, or knockdown of γ-glutamate-cysteine ligase in culture cells, induces apoptosis [84,85]. In this sense it appears interesting to consider GSH depletion as a cellular event preceding (or required for) apoptosis. GSH depletion is in fact an early hallmark in the progression of programmed cell death in response to apoptotic stimuli [86,87]. Indeed GSH depletion has been classically associated to, e.g., a primary increment in ROS production, although GSH depletion may also be dependent on GSH extrusion [88]. Interestingly it has been shown that resveratrol, a plant-fruit-derived polyphenol capable of inducing apoptosis in different experimental models, activates GSH efflux [83]. For instance, GSH efflux-induced intracellular GSH depletion leads to a BAX overexpression-mediated apoptosis activation in lung cancer cells (a ROS-independent mechanism) [83,89,90].

On the other hand, GSH synthesis is upregulated during oxidative stress and inflammation. In practice, oxidants such as ozone, hyperoxia, H_2_O_2_, *etc.* cause short-term falls in intracellular GSH which associate with higher oxidized glutathione (GSSG) levels; this is followed by increases in GSH levels and/or upregulation of γ-glutamate-cysteine ligase mRNA in *in vivo* and *in vitro* models [91-93]. Therefore, oxidants and oxidant-generating systems (if their levels do not compromise cell viability) can upregulate GSH synthesis-linked genes, thus providing paradoxically a protective mechanism against oxidative stress.

Different studies have been tried to elucidate the molecular mechanisms implicated in GSH synthesis regulation [25,57,58,68]. The γ-glutamate-cysteine ligase promoter contains potential cis-acting elements, including consensus recognition sites for binding of different transcription factors such as: activator protein-1 and -2 (AP-1 and AP-2), Sp-1, NF-kB, and electrophile responsive element (ARE) containing an embedded phorbol myristate acetate-responsive element (TRE/AP-1) [94]. These factors are active in response to diverse stimuli and all of them affect the γ-glutamate-cysteine ligase subunit genes. For example, β-naphtoflavone, a planar aromatic xenobiotic, modulates γ-glutamate-cysteine ligase in the liver cell line HepG2 through TRE/AP1 and ARE [95]; in addition, it has been shown that H_2_O_2_-dependent activation of GCL-ARE4 reporter occurs via MAPK pathways without oxidation of cellular GSH or the redox protein thioredoxin-1, thus suggesting that cell GSH/GSSG redox status is not required for regulation of γ-glutamate-cysteine ligase or ARE [96]. Furthermore, antioxidants trigger protection against oxidants either by directly scavenging these molecules or by regulating intracellular GSH levels through the induction of γ-glutamate-cysteine ligase [94]. In fact many antioxidants can exert direct effects upon several signal transduction enzymes independently of their antioxidant function [97].

Nrf-2 is a transcription factor essential in HepG2 cells for the ARE-mediated induction of phase II detoxifying and γ-glutamate-cysteine ligase genes in response to ROS, electrophiles and phenolic oxidants [98]. Mice deficient in Nrf-2 (Nrf-2^−/−^) have shown an increased susceptibility to the injurious effects of hyperoxia, as noted by a marked increase in pulmonary permeability, macrophage inflammation, and epithelial injury as compared to control wild mice [99]. Moreover, reduction in ARE expression and in different redox balance-related enzymes in lung was observed in Nrf-2 ^−/−^ mice *versus* normal mice. In these effects, Nrf-2 participates by directly regulating γ-glutamate-cysteine ligase genes and GSH-dependent enzymes expression [98,100]. Interestingly, Nrf-2 levels have been shown directly associated with resistance to apoptosis [101].

Furthermore, Kelch-like ECH associated protein 1 (Keap-1) plays a critical role in ARE-mediated signaling by down regulating Nrf-2. Keap-1 inhibits Nrf-2 action by binding and retention of the transcription factor in the cytoplasm [102]. Under ROS or electrophilic insults the complex is dissociated and Nrf-2 is translocated to the nuclei where transactivates target genes [94,103].

Nrf-2 expression is also regulated by GSH [94]. Murine embryonic fibroblast survived in the presence of BSO, even though most intracellular GSH was depleted [104], which associated to activated Nrf-2 and up-regulation of antioxidant enzymes. Nrf-2-deficient murine embryonic fibroblasts lost this capacity and ROS accumulation, caspase-3 activation and cell death were promoted. Furthermore, Nrf-2 deficient cells were more susceptible to doxorubicin and BSO treatment-induced cell death than wild cells [94]. Moreover, propyl gallate activated caspases 3, 8, and 9, and induced an increase in p53, Bax, Fas, and Fas Ligand; whereas MAPKs inhibited nuclear translocation of Nrf-2 and induced intracellular GSH depletion in human leukemia [105]. These results showed that an early event of propyl gallate-induced apoptosis is MAPKs/Nrf-2-mediated GSH depletion, thus indicating that Nrf-2 is one of the first factors that induce cell survival under GSH depletion, which points out to this transcription factor as an attractive target in leukemia but also in other cancers sharing similar molecular mechanisms.

The induction of apoptosis through ASK1, under ROS stimulation, is dependent of cellular redox status. Treatment of A549, an established cellular line from human lung adenocarcinoma, with denbinobin induced ROS production and JNK activation with downstream Bim expression. Bim knockdown by siRNA, and *N*-acetyl-L-cysteine or GSH treatment, reduced denbinobin-induced A549 cell apoptosis [106]. In the same way, treatment of hepatoma cells with aloe-emodin, a bioactive anthraquinone of Rheum palmatum with anticancer properties, induced oxidative stress and apoptosis in a mechanism mediated by ASK1 and JNK activation. Furthermore, inhibition of GSH synthesis by BSO, or treatment with glutathione monomethyl ester as a GSH donor, sensitized or protected hepatoma cells to JNK activation. Thus indicating a critical role of oxidative stress and sustained JNK activation in aloe-emodin-mediated apoptotic cell death in human hepatoma cells [107]. On one hand, ASK1 is one of the main activators of apoptosis under oxidative stimuli; whereas on the other hand its activity depends on GSH levels. Therefore modulation GSH levels could be critical to increase the susceptibility of cancer cells to different antitumoral treatments.

### 3.2. GSH Role in Autophagy and Necrosis and the Links between Different Types of Cell Death

Autophagy or programmed cell death type II consists of the selective degradation of cellular components through the lysosomal machinery [47]. This is the major catabolic pathway by which cells degrade and recycle macromolecules and organelles. Autophagy can be activated by different stimuli such as amino acid deprivation, ROS, cancer, pathogen infections, *etc.* [108]. Initially cells use autophagy as a mechanism to preserve their viability. However, if cells cross a critical threshold, macromolecular destruction causes cell death [108]. Autophagy is initiated by the surrounding of cytosolic constituents, macromolecules or organelle, in a closed double membrane structure called autophagosome. Autophagosome fuses with lysosomes to form autolysosomes. The lysosomal hydrolases digest the vesicle content [108]. When stimuli are too high or prolonged, autophagy becomes a prodeath mechanism. Specific morphological changes observed in autophagic cells include a massive vacuolization of the cytoplasm in the absence of chromatin condensation [109]. The role of GSH in autophagy is not fully understood but replenishment of GSH level using *N*-acetyl-L-cysteine prevents autophagy induction and autophagosome formation and protein degradation induced by starvation [110,111]. Lipopolysacharide induces autophagy in a GSH-dependent manner since it is able to increase ROS production and deplete the tripeptide levels [112]. Nitric oxide produces nitrosative and oxidative stress and cellular damage, which induces autophagic cell death in GSH depleted osteoblasts [112-114].

Classically cellular necrosis refers to a cell that, in response to severe physical or chemical damage or a critical decrease in energy availability, swells and explodes, then releases its intracellular content into the surrounding space. Thus, necrosis has been considered an uncontrolled type of cell death [112]. However, there are recent studies suggesting that necrosis is a regulated process involved in multiple development, pathological and physiological scenarios [112]. Inhibition of cellular energy production, generation of ROS, imbalance of cellular Ca^2+^ homeostasis or extracellular cell death signals, are among those stimuli able to induce either apoptosis or necrosis [115]; thus showing that different types of cell death can share, at least in part, common mechanisms. In this sense, time and intensity of stimulus may determine the type of cell death. In fact, depending on GSH depletion and oxidative stress level apoptosis can switch to necrosis [72]. For instance, the sensitivity of Ehrlich ascites tumor cells to TNF-α depends on their GSH content and their rate of proliferation. This is important because tumor cell populations under active proliferative states may show higher GSH levels, and drug- and/or radiation-resistant tumors have increased cellular levels of GSH. In fact, TNF-α induces a shift towards oxidation in the mtGSH status, a fact that is consistent with the hypothesis that mtGSH plays a key role in scavenging TNF-α-induced ROS [116].

## 4. Glutathione Depletion and Cancer Therapy

### 4.1. Potential Benefits and Limitations

The fact that GSH depletion can be deleterious for cancer cells and, potentially, enhance the effectiveness of chemotherapy and/or ionizing radiations, is known [7,25,56]. The value of GSH depletion in sensitizing tumor cells to ionizing radiation was first demonstrated in several human lymphoid cell lines [117]. Indeed, as reviewed later, cancer cells containing low GSH levels (<10% of their control values) were found much more sensitive than control cells to the effects of γ-irradiation [2]. GSH and drug resistance of cancer cells have been also linked [25,118]. Chemoresistance is a multifactorial phenomenon and many studies show that a coordinated expression of efflux transporter proteins and phase II conjugating enzymes in tumor cells is linked to the development of the multidrug resistance phenotype. In particular, the overexpression of GSH transferase and efflux pumps in tumors may reduce the reactivity of various anticancer drugs.

The relationship between GSH depletion, chemotherapy, and/or the radiation response has been examined in many tumor cells after treatment with different drugs, including BSO, diethylmaleate, 2-oxothiazolidine-4-carboxylate, and different radiosensitizing agents [2,8,25,119,120]. However BSO (as well as other thiol-depleting agents) is non-specific and, besides promoting tumor GSH depletion *in vivo*, can cause irreversible damage in most normal tissues. Moreover, GSH depletion only appears to be therapeutically effective when very low levels of this tripeptide can be achieved within the cancer cells [25]. Thus, achievement of selective tumor GSH depletion under *in vivo* conditions appears as a superb pharmacological challenge.

In fact, since the molecular background was firmly established, the potential benefits of GSH depletion for cancer therapy have remained in the shadow for two decades. Nevertheless, recently, new research has offered some light on how to make GSH depletion a useful tool in cancer therapy.

### 4.2. Molecular Mechanisms Channeling Cytosolic GSH Efflux

Drug resistance frequently associates with over-expression of P-glycoprotein and/or multidrug resistance proteins (MRPs), which work as pumps extruding cytotoxic drugs out of the tumor cells [24,121-123]. Efflux of GSH, GSSG, and GSH S-conjugates (xenobiotics or metabolites) from different mammalian cells appears channeled through different MRPs [24,25,124-127]. Potential endogenous substrates for MRPs have been recently revised by Ballatory *et al.* (2009) [126,128]. A total of nine multifunctional mrp genes have been identified (from mrp1 to mrp9). But MRP-1, MRP-2 and MRP-4 are principally responsible for the transport of GSH [127,129,130], whereas the others MRPs have different substrates (e.g., bilirubin glucuronosides for MRP2 and MRP3, or cyclic AMP and cyclic GMP for MRP4, MRP5, and MRP8) [130]. Activation of Nrf2-Keap1 pathway stimulates the induction of MRPs expression, as well as genes responsible for both the synthesis and conjugation of GSH [129,131,132].

MRP1, the first to be cloned, is overexpressed in many drug-resistant cancer cells and functions as a drug efflux pump [127]. Its expression is much lower in most normal tissues where it works to decrease accumulation of xenobiotics and their metabolites [127,133]. MRP1 mediates the ATP-dependent transport of different organic (including naturally occurring conjugated metabolites and most GSH-, glucuronide-, and sulfate-conjugated products of phase II xenobiotic metabolism) and inorganic anions (e.g., antimonial and arsenical oxyanions) [133]. MRP1 may even act in cooperation with GSH transferases (e.g., GSTP1) to protect cancer cells from cytotoxic drugs [134].

GSH depletion by BSO-induced inhibition of γ-glutamate-cysteine ligase [26] resulted in a complete reversal of resistance to anticancer drugs of different cell lines overexpressing MRP1 but had no effect on P-glycoprotein-mediated multidrug resistance [135]. Cancer cells may release GSH through MRP1 even in the absence of cytotoxic drugs [124]. In addition GSH is required for the transport by MRP1 of GSH conjugates of, e.g., vincristine or doxorubicin, but is also evident that in some cases (e.g., vinca alkaloids) drugs are co-transported with GSH [127]. This requirement for GSH may explain why drug extrusion through MRP1 decreases in BSO-treated cells, thus indicating that MRP1 also transports non-conjugated GSH [136,137]. MRP1-channelled GSH export from cells can be also increased by different xenobiotics, including arsenite, verapamil (VRP), and some naturally-occurring flavonoids [138,139], although for these molecules there is no evidence that their MRP1-mediated transport could be stimulated by GSH. On the other hand, there is reciprocal cooperativity between GSH and, e.g., vincristine in their MRP1-mediated co-transport [140]. Moreover GSH increases: (a) the inhitory effect of, e.g., flavonoids and vinca alkaloids on the MRP1-mediated transport of organic anions; whereas (b) stimulates the transport of different glucuronate, sulfate and GSH conjugates [127]. Nevertheless, it remains undefined what exactly determines the GSH requirement for the transport of a particular substrate, although it appears plausible that MRP1 may have several drug-binding sites.

We have presented evidence showing that GSH is released from highly metastatic B16-F10 melanoma cells through MRP1 and the cystic fibrosis transmembrane conductance regulator (CFTR) [141]. The CFTR, a member of the ABC family of membrane transport proteins with structural similarities with MRP1, forms a phosphorylation- and ATP-dependent channel permeable to Cl^−^ and to other larger organic anions, including GSH [142]. The CFTR, which is expressed in different cell types, is activated by the binding of ATP to its cytoplasmic nucleotide-binding domain and by phosphorylation of key Ser residues in the regulatory domain [143]. Phosphorylation is mediated principally by cAMP-dependent PKA and by phosphokinase C (although to a lesser degree than the activation by PKA) [144]. Basal expression of the CFTR gene is dependent on PKA activity [145]. Treatment of human colon carcinoma T84 cells with the PKA-selective inhibitor *N*-[2-(p-bromocinnamylamine) ethyl]-5-isoquinolinesulfonamide (H-89) causes a complete suppression of CFTR gene expression without affecting other constitutively active genes [145]. Addition of anti-CFTR antibodies to MRP^−/−^1 (MRP 1 knockout) B16-F10 cells practically abolished GSH efflux from B16-F10 cells [141]. This indicating the existence of different molecular channels controlling the rate of GSH efflux from B16-F10 cells: MRP1, and the Bcl-2-dependent CFTR transporter [25]. Nevertheless, the possibility cannot be ruled out that other mechanism(s) may also be working in other cell types or that different CFTR gene mutations could be found when comparing different cancer cells. Although what appears really relevant is the fact that the rate of efflux may become an important factor regulating intracellular GSH content and, thereby, cancer cell survival and resistance to treatments.

We showed that Bcl-2 antisense oligodeoxynucleotides (Bcl-2-AS; which prevent the Bcl-2-dependent inhibition of GSH efflux through CFTR) and VRP (which increases the MRP1-dependent GSH efflux) independently increased rates of GSH efflux from perifused B16M-F10 cells [141]. The perifusion technique through a chamber, where isolated cancer cells are maintained in suspension at 37 °C, represents an experimental setup that mimics *in vivo* conditions by providing a constant supply of glucose, amino acids, oxygen, CO_2_ and GSH at physiological plasma concentrations. This setup allowed us to use a VRP concentration (1 μM) that corresponds to clinically accepted and nontoxic levels in plasma [141]. Intracellular GSH content was significantly decreased (∼30%), as compared to controls, in Bcl-2-AS- and VRP-treated B16M-F10 cells after 6 h of perifusion. However, at 12 h of perifusion, GSH levels were ∼70% higher in Bcl-2-AS- and VRP-treated B16M-F10 cells than in controls [141] Thus, it appears that loss of GSH accelerates their rate of intracellular synthesis. In fact, we found that increased GSH efflux was associated with γ-glutamate-cysteine ligase overexpression [141].

In growing tumors, cyst(e)ine, whose concentration in blood is low, may become rate-limiting for GSH synthesis and cell growth [17,146]. Cystine is predominant outside the cell since Cys rapidly autoxidizes to cystine in the extracellular fluids, whereas once it enters the cell through the Xc^−^ system, cystine is reduced to Cys and used preferentially for protein and GSH synthesis [147].

Tumor γ-glutamyl transpeptidase (GGT) activity (frequently overexpressed in many tumors [148]) can cleave extracellular GSH (the liver being the main exporter), releasing γ -glutamyl-aminoacids and cysteinylglycine, which is further cleaved by membrane-bound dipeptidases into Cys and Gly [26]. Free γ-glutamyl-amino acids, Cys, and Gly entering the cell serve as GSH precursors [26]. Therefore, GGT may provide tumor cells with a growth advantage at physiological concentrations of cyst(e)ine [17,146].

Therefore, if the increase in GSH content, which follows the effect of Bcl-2-AS and VRP on GSH efflux, depends on cyst(e)ine availability, and if this is provided in part by GGT, then inhibition of the activity of this enzyme could limit GSH synthesis. This possibility was tested by adding acivicin (ACV), an irreversible GGT inhibitor [149], to the B16 perifusion system. ACV decreased GGT activity to non-detectable levels and decreased GSH synthesis but without affecting the rate of GSH efflux or the rate of cystine uptake [141]. Hence, Cys availability within malignant B16M cells appears, indeed, to be modulated by its GGT-dependent generation from extracellular GSH. In consequence, we concluded that Bcl-2-AS- and VRP-induced acceleration of GSH efflux, combined with inhibition of GGT, could represent a useful methodology to deplete GSH in B16-F10 cells *in vivo.* Importantly this approach appeared selective since it did not decrease GSH in normal tissues [17].

### 4.3. Mitochondrial GSH and the Glutamine Trap

Mitochondrial dysfunction is a common event in the mechanisms leading to cell death [150]. Mitochondrial permeability transition is critical in the process leading to apoptosis, and it is linked to the opening of a permeability transition pore complex [150] (see also above). This molecular gate is regulated by many endogenous factors, including divalent cations (e.g., Ca^2+^ and Mg^2+^), protons, the concentration of adenine nucleotides, the thiol (controlled by GSH) and the pyrimidine redox state, the rate of ROS and reactive nitrogen species generation, the concentration of lipoids (e.g., ceramide), the concentration of certain peptides targeting proteins for mitochondrial import, and the function of different pro- and anti-apoptotic proteins [151].

GSH, which is not synthesized by mitochondria but taken up from the cytosol through a multicomponent transport system [2,86], is involved in the defense against peroxides generated from the electron transport chain [152] and is an important regulator of the mitochondrial permeability transition and permeability transition pore opening [11,25,150,151]. Thus, impairment of GSH uptake by mitochondria (or direct mtGSH depletion) appears a useful mechanism to sensitize malignant cells to molecular effectors (e.g., oxidative stress inducers and/or cytotoxic drugs) capable of activating the mitochondrion-based death mechanism. Naturally, to be really effective under *in vivo* conditions, such an approach should be totally (or at least partially) selective for the cancer cells and therefore not affect normal cells.

Tumor cells need a constant supply of both energy and nitrogen substrates. Having originated from Warburg's [153] seminal observation of aerobic glycolysis in tumor cells, most of this attention has focused on glucose metabolism. However, since the 1950s cancer biologists have also recognized the importance of L-Gln, the most abundant amino acid in blood, as a tumor nutrient [154]. L-Gln, which accounts for 50% of the whole body pool of free amino acids, contributes to essentially every core metabolic task of proliferating tumor cells: it participates in bioenergetics, supports cell defenses against oxidative stress and complements glucose metabolism in the production of macromolecules [155]. Indeed, many tumors and rapidly dividing cells exhibit a remarkable preference for L-Gln as respiratory fuel [156]. In many cancer cells, L-Gln is the primary mitochondrial substrate and is required for maintenance of mitochondrial membrane potential and integrity and for support of the NADPH production needed for redox control and macromolecular synthesis [157]. A host to tumor net flux of L-Gln in mice bearing Ehrlich tumors has been described [158], and studies in different tumors have demonstrated that L-Gln utilization correlates with: (a) increased mitochondrial phosphate-dependent glutaminase activity that is dependent on Rho GTPases and NF-kB activity [155,156,159]; and (b) oncogenic levels of Myc which induce a transcriptional program that promotes glutaminolysis and trigger cellular addiction to L-Gln as a bioenergetic substrate [160]. Tumors with high rates of glutamine uptake and metabolism can behave as glutamine traps, depleting host glutamine stores, producing glutamate rapidly, and resulting in cachexia [156,161]. However, we recently suggested that these peculiarities could represent an advantage in terms of developing a novel strategy to treat cancer.

Providing supplemental L-Gln during cancer treatment, by reducing the incidence of gastrointestinal, neurological and possibly cardiac complications, has the potential to minimize chemotherapy- and/or radiation-related toxicity [162-166]. L-Gln, preferentially formed and stored in skeletal muscle, is the principal metabolic fuel for small intestine enterocytes, lymphocytes, macrophages, and fibroblasts [167]. L-Gln-enriched diets (GED) may support muscle L-Gln metabolism without stimulating tumor growth [159,162-167]. Moreover, a high rate of L-Gln oxidation may render the mitochondria more susceptible to ROS-mediated cytotoxicity by TNF-α [11,168]. In previous studies in Ehrlich ascites and highly metastatic B16-F10 melanoma tumor cells, we found that L-Glu derived from L-Gln competitively inhibited GSH transport into mitochondria [159,169], thereby depleting selectively tumor mtGSH under *in vivo* conditions [159] and rendering tumor cells more susceptible to oxidative stress-induced mediators, such as TNF-α [11].

We developed an original methodology to produce L-Gln-adapted malignant cells with very high metastatic potential (B16M-F10-Gln^+^). These cells grew as a local tumor in the footpad of mice fed a GED which, as compared with control tumor-bearing mice fed an standard diet, did not accelerate cancer growth [169]. Moreover, the GED significantly decreased the loss of body weight and the release of skeletal muscle L-Gln [169], which suggests anticachectic properties. Indeed, in randomized, double-blind controlled clinical trials, cancer patients receiving L-Gln-supplemented parenteral nutrition had improved nitrogen balance, a diminished incidence of clinical infections and less extracellular fluid accumulation, clinical facts that are consistent with the potential role of L-Gln in stimulating protein synthesis in skeletal muscle, supporting endothelial function and integrity, and enhancing immune functions (see [169] and references therein for a review). Furthermore, in rats, provision of a GED during whole abdominal irradiation (10 Gy, a dose that results in a 50% mortality rate in a few days) exerted a protective effect on the small bowel mucosa by supporting crypt cell proliferation and, thereby, accelerated healing of the irradiated bowel and reached a 100% survival of the irradiated animals [162,164]. Besides, L-Gln supplementation may also decrease the incidence and/or severity of chemotherapy-associated mucositis, irinotecan-associated diarrhea, paclitaxel-induced neuropathy, hepatic veno-occlusive disease in the setting of high dose chemotherapy and stem cell transplantation, and the cardiotoxicity that accompanies anthracycline use [164]. Studies in patients receiving L-Gln-enriched nutrition for several weeks confirmed the clinical safety of this approach in a catabolic patient population [163]. Therefore, supplementation of a GED appears a feasible methodology to deplete mtGSH levels [10,25,159,169] in growing malignant cells, and in addition may promote beneficial effects for the cancer patient.

## 5. GSH Depletion: A Feasible Strategy to Improve Cancer Therapy

**Figure 2.**
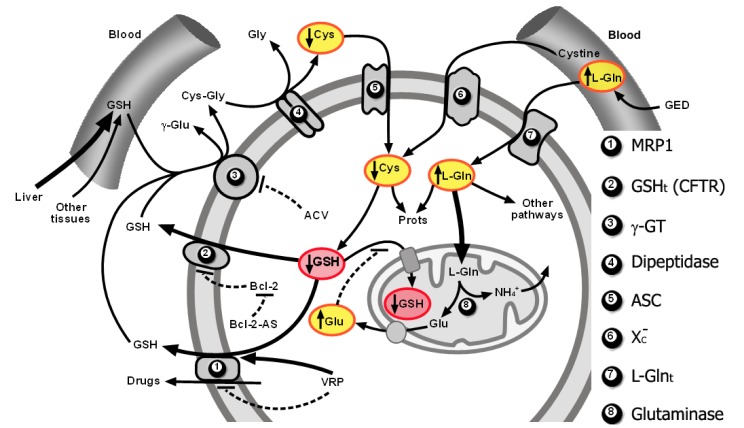
Experimental therapy to deplete GSH levels in cancer cells. Increased cytosolic GSH efflux, Cys shortage-induced decrease in GSH synthesis and inhibition of GSH transport into mitochondria, lead to cytosolic and mitochondrial GSH depletion, thus sensitizing tumor cells to chemo- and radiotherapy. Cytosolic GSH efflux is stimulated by verapamil and Bcl-2 antisense oligodeoxynucleotides treatment. Acivicin-induced γ-GT inhibition limits Cys availability for GSH synthesis. L-Glu is a competetive inhibitor of GSH transport into mitochondria. High levels of cytosolic L-Glu are achieved by a glutamine-enriched diet which promotes a higher mitochondrial glutaminase activity. This strategy may render cancer cells (and abnormal cells) more sensitive to oxidative stress inducers and cytotoxic drugs.

As explained above, GSH efflux activation through MRP1 and a putative GSH transporter of an undefined molecular nature that was found to correspond to CFTR in B16 melanoma cells, limitation of Cys supply for *de novo* synthesis of GSH, and glutamate (Gln-derived)-induced competitive inhibition of cytosolic GSH transport into mitochondria, can promote both cytosolic and mitochondrial GSH depletion in the tumor cells. This strategy, which preferentially affects tumor and not normal cells, causes a decrease in tumor cell defenses and resistance to oxidative stress inducers (such as TNF-α or ionizing radiations) and cytotoxic drugs [10,11,17,25,141,159,169]. Figure 2 schematically summarizes the molecular interrelationships leading to depletion of the cytosolic and mitochondrial pools.

Nevertheless, it is essential to keep in mind that *in vivo* growing tumors represent a heterogeneous tissue, with different cell types (cancer, blood and lymphatic vessels, fibroblasts, immune cells, *etc.*) and tissue-specific microenvironments. In addition, tumor cells are also heterogeneous and may contain several cell subsets with different levels of resistance to death-inducing stimuli. Regarding GSH, its content within the cancer cell may vary depending on the rate of cell growth. In addition, we cannot rule out the possibility that mechanisms channeling GSH efflux differ when comparing different cancer types, or that, e.g., different CFTR gene mutations, could be found when comparing different cancer cells.

What is then the good news? This approach is the first to achieve selective GSH depletion in different cancer cell types growing *in vivo*. Drugs used all function at clinically acceptable doses.

VRP (activating GSH efflux through MRP1), is a calcium ion influx inhibitor frequently used for the management of hypertension and angina pectoris, which has also been used in patients receiving chemotherapy for example, myeloma or acute lymphocytic leukemia. Cells isolated from these patients showed increased accumulation of daunorubicin or vincristine [170] when patient plasma concentrations of VRP were similar to murine VRP plasma levels in our experiments [141].

ACV (a GGT inhibitor) has been evaluated for antitumor activity in phases I and II clinical trials because expression of GGT was observed in melanoma and cancers of the liver, lung, breast, and ovary [148,171]. These trials revealed central nervous system toxicities, so a maximum dose of 50 mg ACV/m^2^/day in combination with the amino acid solution aminosyn to reduce ACV uptake in the central nervous system was proposed. However, the application of ACV described here is not predicted to require aminosyn. The pharmacokinetic parameters of ACV in patients [171], our efficacy results, and previous preclinical studies [141] all suggest that ACV plasma levels sufficient to block GGT activity in tumors would be below the levels that resulted in central nervous system toxicity.

Bcl-2-AS therapy has been evaluated in multiple clinical trials [172-174]. Bcl-2-AS therapy using G3139, for example, an 18-base phosphorothioate oligonucleotide complementary to the first six codons of the Bcl-2 mRNA, selectively and specifically inhibits Bcl-2 expression and promotes apoptosis in different human and murine cancer cell lines [175]. G3139 is well tolerated at doses comparable to that used in our studies (e.g., [176] and references therein). Systemic administration of G3139 to Shionogi tumor-bearing mice led to a rapid decrease of tumor size (higher when chemotherapy was simultaneously administered), whereas the oligonucleotide did not affect Bcl-2 expression in normal organs [177,178]. G3139-induced tumor regression without dose-limiting toxicity was also observed in other tumors, melanoma, lymphoma, or gastric cancers for example [175]. Furthermore, synergism of the G3139 and anticancer drugs was also shown in different tumors [172,179-182].

GEDs are used clinically (see above) and, thus, tumor cell adaptation to a GED (as a previous step before cytotoxic/target therapies are applied) is feasible.

Therefore the proposed strategy (or variations of it) can easily be applied and may represent a significant improvement in the therapy of different cancers. With this aim, further experimental work and clinical trials, combining GSH depletion with current cancer therapy protocols, are necessary steps forward.

## 6. Conclusions

GSH participates in cancer cell protection against xenobiotics, ionizing radiations, and oxidative stress-inducing biotherapy. In addition mitochondrial GSH oxidation, in particular, favors opening of the mitochondrial permeability transition pore complex, thus facilitating the release of death-related molecular signals. Moreover, GSH is also involved in regulating other types of cancer cell death, including necrosis and autophagy.Identification of the mechanisms controlling GSH homeostasis and fluxes in cancer cells allowed to elucidate a potential, GSH-depletion-based, strategy to improve the efficacy of cancer therapy. This involves (a) cytosolic GSH depletionthrough an increase of GSH efflux out of cells; and (b) mitochondrial GSH depletion through inhibition of its transport into these organelles. Nevertheless these mechanisms need to be assayed in different tumor types in order to make this strategy as general as possible.

## Abbreviations

GSH: Glutathione
ROS: Reactive oxygen species
TNF: Tumor necrosis factor
ARE: Antioxidant response elements
MAPK: Mitogen-activated protein kinase
BSO: Buthionine sulfoximine
mtGSH: Mitochondrial GSH
GSSG: Oxidized GSH
MRP: Multidrug resistance protein
ABC: ATP-binding cassette
CFTR: Cystic fibrosis transmembrane conductance regulator
PKA: Protein kinase A
VRP: Verapamil
Bcl-2-AS: Bcl-2 antisense oligodeoxynucleotides
GGT: γ-glutamyl transpeptidase
ACV: Acivicin
GED: l-Gln enriched diet
